# Investigating the Effect of Wire Drawing and Heat Treatment on the Response of Ni_50.9_Ti_49.1_ R-Phase Actuators

**DOI:** 10.3390/ma18214931

**Published:** 2025-10-28

**Authors:** Josephine Ryan Murphy, Muhannad Ahmed Obeidi, Inam Ul Ahad, Dermot Brabazon

**Affiliations:** 1 School of Mechanical and Manufacturing Engineering, Dublin City University, 9 Dublin, Ireland; obeidimuhannad@gmail.com (M.A.O.); inamul.ahad@dcu.ie (I.U.A.); dermot.brabazon@dcu.ie (D.B.); 2 I-Form Advanced Manufacturing Centre Research, Dublin City University, 9 Dublin, Ireland; 3 Advanced Metallic Systems Centre for Doctoral Training, School of Mechanical & Manufacturing Engineering, Dublin City University, 9 Dublin, Ireland

**Keywords:** shape memory effect, heat treatment, nitinol, actuators, cold work, R-phase

## Abstract

In this investigation, Ni_50.9_Ti_49.1_ wires cold rolled to 40% and straight annealed at 480 °C, 510 °C, and 550 °C, respectively, were heat treated to shape set these wires as helical springs and enhance their SME for use as electro-mechanical actuators. These spring actuators were heat treated at 350 °C, 400 °C, and 450 °C for 30, 60, and 90 min. The wires’ performance as actuators was assessed on a custom-built testing rig, which measured both the stroke and actuation time for each wire. Additionally, the wires were characterised experimentally by DSC, XRD, and nanoindentation. The final resulting properties of the R-phase transformation helical spring actuator are controlled by the competing mechanisms of dislocation annihilation, and precipitation of Ni_4_Ti_3_, as well as the prior thermomechanical treatment. The optimum conditions for actuator response in Ni_50.9_Ti_49.1_ 40% cold-worked wires were a straight annealing temperature of 480 °C and shape-setting aging conditions of 450 °C for 60 min. These parameters result in the optimum combination of defect annihilation and density of precipitates, resulting in a high-stroke (56 mm), low-hysteresis (2.68 °C) actuator with an actuation time of 6 s.

## 1. Introduction

Nitinol (NiTi) is a nickel–titanium shape memory alloy (SMA) of equiatomic composition. SMAs are alloys that exhibit two unique mechanical properties known as superelasticity (SE) and shape memory effect (SME) [1]. SE and SME are achieved due to a reversible, thermally activated transformation between the two main crystal phases of NiTi: a high-temperature austenitic phase (B2) with BCC crystal structure, and a low-temperature martensitic phase (B19’) with monoclinic lattice structure [2,3]. SE also refers to the material’s ability to recover strains isothermally during a mechanical load/unload cycle without acquiring any residual stresses, while SME is defined by the material’s ability to recover large deformations, returning to its original shape upon heating above a transition temperature [4].

While SE and SME have been found to exist in many different alloys, NiTi is the most popular shape memory alloy (SMA) owing to its high tensile strength, corrosion resistance, and biocompatibility [5]. As a result, NiTi has numerous applications across a wide range of industries generally availing of either SE or SME. For example, the biomedical industry makes use of NiTi’s SE along with biocompatibility and corrosion resistance for devices such as catheters and stents; meanwhile, the aerospace industry takes advantage of the SME, high force-to-weight ratio, and spark-free response to thermal stimuli for use as sensors and actuators [4,6,7]. The prevalence of either an SE or SME characteristic depends on the composition of the alloy, which in turn determines the phase. A higher titanium content results in higher transformation temperatures (TTs), a martensitic phase, and an SME response, while a higher nickel content results in lower TT, an austenitic phase, and an SE response [8]. For example, Wen et al. [9] found that NiTi samples produced from powder with a Ni content of 50.73 at.% displayed SME, while NiTi with Ni content 50.93 at.% and 51.27 at.% displayed SE and had A_f_ temperatures of 55 °C, 31 °C, and 25 °C, respectively. 

Although Ti-rich NiTi is generally used for SME, equiatomic or Ni-rich NiTi is more widely available and can–through thermomechanical processing–be used for SME applications. Ni-rich NiTi also shows reduced thermal hysteresis, which is an important factor for actuators, allowing actuator activation and de-activation to occur within a smaller temperature, the mechanical properties of NiTi are dependent on the temperature of the operative environment relative to the transformation temperatures for the alloy range [10,11]. This means that to be able to tailor NiTi SMA actuators to a wide range of applications, SME Ni-rich NiTi must also be investigated. 

As well as chemical composition, the processing routes of NiTi wire also drastically impact the material and mechanical properties. NiTi wires are produced by a thermomechanical process known as wire drawing, which involves a series of cold rolling through dies to reduce the diameter and inter-pass annealing, followed by straight annealing at various temperatures and times to control the final properties of the wire. The cold-working process introduces lattice defects and randomly distributed dislocations into the microstructure, which can result in increased strength and reduce SME while annealing, depending on the annealing parameters, can rearrange or annihilate the dislocations as well as cause the formation and dissolution of precipitates such as Ni_4_Ti_3_, thereby altering the chemical composition of the matrix. The thermo-mechanical history is therefore instrumental in determining the material’s properties [12,13].

The nanocrystalline grain structure formed due to cold work can impede B19’ nucleation, resulting in the formation of the intermediate R-phase. Additionally, the strain fields introduced by precipitates such as Ni_4_Ti_3_ can further stabilise the R-phase [14]. The R-phase can be beneficial for sensor and actuator applications due to its increased cycling stability and low temperature hysteresis, allowing it to operate in a smaller temperature range [15,16]. A trade-off from the R-phase is the small recoverable strains (<1%) compared to the martensite transformation strain (6%) However, the R-phase transformations can still be effectively used as SMA actuators when used in the form of a helical spring, allowing a much larger stroke per unit of length of the actuator and adequate work output despite the low recoverable strains [17,18]. In addition to having larger strokes, linear SME helical spring-based actuators are more compact than wire actuators [19].

Shape setting is performed by holding the material in the trained shape at an aging temperature for a specific duration, followed by quenching to entrain the higher austenite-phase structure. Studies have also found that constraining NiTi for shape setting increases the effect of heat treatment on the material. Favier et al. [20] found that aging has a stronger influence on the transformation and mechanical behaviour of Ni_50.8_Ti_49.2_ in constrained samples, indicating faster aging kinetics. This is attributed to the interaction between internal stresses from Ni_4_Ti_3_ precipitates and the externally applied stress during constrained aging.

Lenticular Ni_4_Ti_3_ precipitates form coherently with the matrix, generating anisotropic stress fields with compressive stress perpendicular to the precipitates. When an external stress is applied, the precipitates align preferentially with the external tensile stress to relieve internal stresses, perpendicular to the internal compressive stress. During constrained shape setting, NiTi undergoes a phase transformation to the B2 phase, attempting to recover its straight-annealed shape. However, the applied constraint prevents full recovery, leading to stress buildup, potential plastic deformation, and pinning of reoriented martensite, which hinders transformation and reduces shape recovery. Benafan et al. [21] found that heating a 10 mm rod of Ni_49.9_Ti_50.1_ at a rate of 15 °C min^−1^ relaxed these stresses, ultimately reaching zero stress at 450 °C. They also noted that isothermally holding the sample at a lower temperature above A_f_ for a period of time can also result in complete stress relaxation. Liu et al. [22] found that shape setting Ni_50.7_Ti_49.3_ 0.5 mm wire at temperatures above 450 °C showed negligible improvements in shape recovery compared to samples shape set at 450 °C for 60 min, and increased the risk of oxidation. Therefore, in this study, shape setting is performed at 350 °C, 400 °C, and 450 °C for 30, 60, and 90 min.

Much of the current literature focuses on the effect of wire drawing steps on the material characteristics of NiTi wire, as well as the influence of heat treatment on the R-phase. There is little research on the effect of the thermomechanical processing of NiTi wires on the functional properties of Ni-rich SMA actuators based solely on R-phase transformations. While there have been many studies on the effects of the degree of cold work and heat treatment on Ni-rich nitinol, they tend to focus on the SE [23]. In the case where SME is studied in Ni-rich nitinol, the actuator performance is not directly assessed but instead tested by methods such as the bend and free recovery test, which does not allow the measurement of properties such as actuator stroke [24,25]. Finally, in the case where the functional performance of SMA actuators is assessed using setups such as linear actuators, the materials used are Ti-rich NiTi [26,27]. This study aims to address gaps in the literature related to the actuation performance of Ni-rich R-phase actuators. In particular, it studies the effect of different wire-drawing histories and shape-setting parameters on the stroke and response times of NiTi helical springs. Ni_50.9_Ti_49.1_ wires from the same ingot with different thermomechanical histories were constrained and shape set as helical springs to investigate the effect of straight annealing and shape-setting parameters on actuator performance. Actuator performance, including stroke and actuation time, was measured and is reported herein using an in-house built rig, and the properties of the wires were characterised using DSC, XRD, SEM, and nanoindentation.

## 2. Materials and Method

### 2.1. Materials and Sample Preparation

In this study, three distinct nitinol wires (denoted as Wire 1 (W1), Wire 2 (W2), and Wire 3 (W3)) are fabricated using traditional wire-drawing and straight-annealing methods and supplied by Fort Wayne Metals, Castlebar, Co., Mayo, Ireland. All three wires originate from the same ingot material source with a composition of 50.90 atomic percent nickel (at.% Ni) and an ingot A_f_ temperature of −5.5 °C tested according to ASTM E2994 and ASTM F2063, respectively. All three wires were cold worked to 40%, resulting in a diameter of 0.46 mm and an ultimate tensile strength (UTS) of 1450 MPa. During the straight annealing step, W1, W2, and W3 were annealed at 480 °C, 510 °C, and 550 °C, resulting in active austenite finish temperatures of 6.37 °C (W1), 23.11 °C (W2), and 44.58 °C (W3), respectively, as measured by DSC analysis in this study. The characterisation equipment utilised in this work is detailed in Section 2.4. For clarity, the active austenite finish temperatures of the as-received wires are herein referred to as A_f0_ temperatures. 

### 2.2. Heat Treatment and Shape Setting

A design of experiments (DoE) model with three parameters at three levels, as can be seen in Table 1, was employed to generate 27 distinct process parameter combinations. The complete set of process parameters, along with their respective sample numbers, is provided in Table 2. To ensure repeatability, three samples (n = 3) were prepared for each set of parameters, resulting in a total of 81 fabricated and tested samples.

From each of the three wires, 27 sections, each measuring 200 mm in length, were cut. To shape these sections into spring actuators, a helical thread with a diameter of 10 mm and a pitch of 1 mm was used as a mandrel. Each sample was mechanically fixed at one end and then wound around the mandrel within the thread groove. Six full rotations around the mandrel following the threaded groove were completed, and subsequently, the wire was mechanically fixed at each end to maintain the spring configuration. The samples were then placed in a box furnace while attached to the threads, enabling simultaneous shape setting and aging treatments. The samples were heat treated in a box furnace at 350 °C, 400 °C, and 450 °C for 30, 60, and 90 min. Upon removal from the furnace, the samples were immediately quenched in water to establish a clear finish time and prevent further aging. 

### 2.3. Stroke and Actuation Time

**Figure 1:**
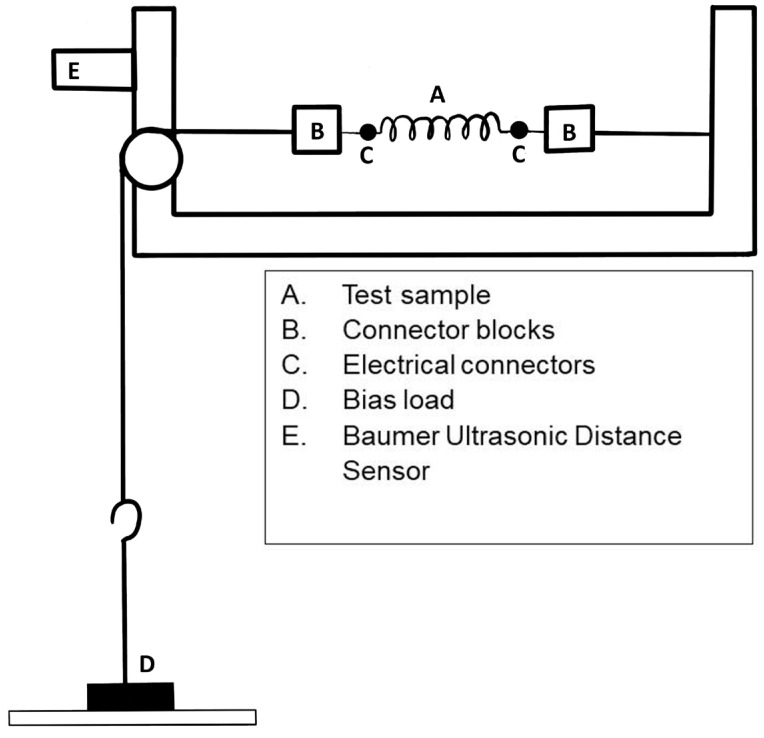
Schematic of in-house designed and built actuator testing rig.

After heat treatment, the samples were removed from the threads and loaded onto the in-house-built NiTi actuator testing rig. A schematic of this experimental rig is shown in Figure 1, where the spring actuator ‘A’ is attached to the stationary wall of the testing rig on one side and connected to the bias load ‘D’ on the other via nylon wire, joined using floating connector blocks ’B’. The bias load, of mass 200 g, hangs from the nylon wire attached to the actuator while a Baumer Ultrasonic Distance Sensor (Model: U300.D50-DPMJ.72N, Baumer International GmbH, Stockack, Germany) ‘E’ was employed to measure the displacement of the sample. To initiate the Shape Memory Effect (SME) and lift the bias load, a current of 2 A was applied across the actuator through electrical connections at ‘C’ for 10 s. A Fluke RSE600 Infrared Camera was used to record the temperature of the sample, and a LabVIEW 2021 program was used to control the current and to measure the data from the camera and displacement sensor. The experiments were carried out according to the steps below:

- The sample is loaded onto the rig at ‘A’ and attached on either end by the connector blocks ‘B’.
- Electrical connectors are attached at both ends of the sample at ‘C’.
- The bias load ‘D’ is attached, causing the sample to stretch.
- Using a LabVIEW program, a current of 2 A is applied to the sample via the electrical connectors.
- The application of current causes the sample to contract and lift the bias load.
- The distance sensor ‘E’ connected to the LabVIEW program records the change in distance between the sensor and the bias load w.r.t time.
- The thermal camera connected to the LabVIEW program records the temperature of the sample w.r.t time during heating.
- After 10 seconds, the current switches off and the sample is left to cool.
- The distance sensor and thermal camera continue to record data as the sample cools and stretches, lowering the bias load.
- The data from the displacement sensor is exported from the LabVIEW program and used to calculate the maximum stroke and the actuation.

**Figure 2:**
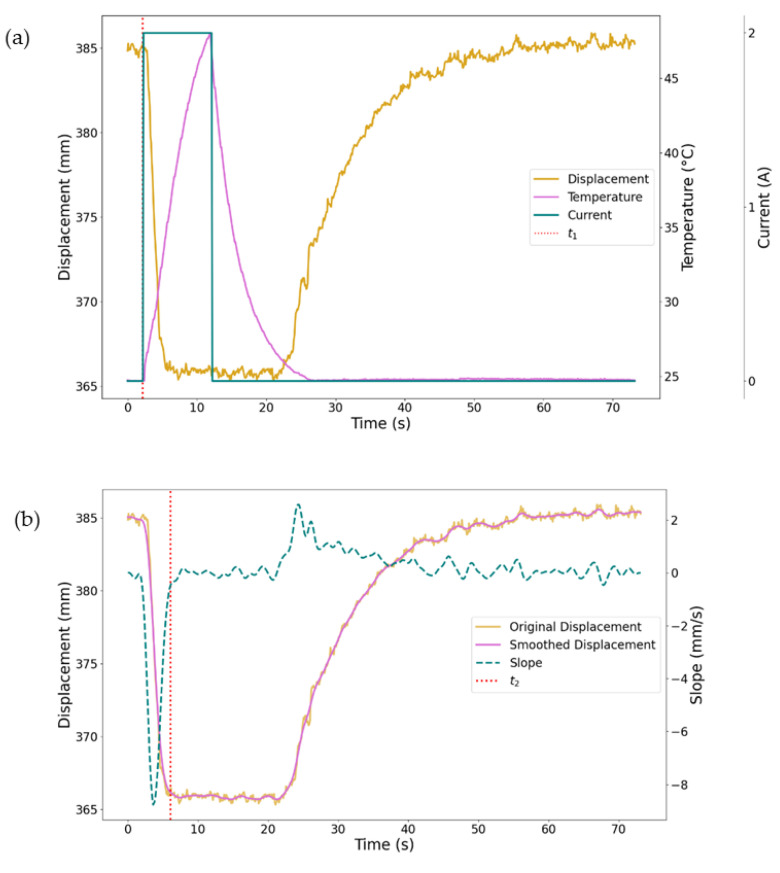
(**a**) Plot of the displacement, temperature, current, and t_1_ from the LabVIEW data for sample 1. (**b**) Plot of the original displacement, the smoothed displacement, the slope of the smoothed displacement, and t_2_.

This test process was repeated on each wire three times (n = 3). The data exported from LabVIEW were used to plot the graph in Figure 2a. To calculate the stroke and actuation time, the noise from the displacement sensor is reduced using a Gaussian distribution function with gamma = 5. From the smoothed displacement, the slope of the displacement curve is calculated, resulting in the curve in Figure 2b. Stroke is defined as Stroke = s_1_ − s_2_, where s_1_ is the distance between the bias load and the displacement before current is applied and s_2_ is the distance between the bias load and the displacement sensor after a current of 2 A has been applied for 10 s. Actuation time (t_a_) is then defined as t_a_ = t_2_ − t_1_, where t_1_ is the time at which the current is switched on and t_2_ is the time at which the slope of the smoothed displacement curve is less than −0.5. 

### 2.4. Material Characterisation

Differential Scanning Calorimetry (DSC) analysis was conducted using a TA Instruments® Discovery DSC2500 instrument (±0.025 °C, TA Instruments®, New Castle, DE, USA) to determine the transformation temperatures (TTs) of both the as-received and heat-treated wires. The wire samples for DSC were prepared by cutting the sample into sections using wire cutters and placing them into aluminium pan crucibles. These crucibles were then positioned in the DSC and prepared for testing by ramping the temperature to 100 °C at a rate of 100 °C/min and then holding isothermally for 5 min. The tests were then carried out by cooling the samples to −150 °C at a rate of 10 °C/min, followed by an isothermal hold for 5 min, and subsequently heating to 150 °C at a rate of 10 °C/min.

X-ray Diffraction (XRD) tests were performed using the PANalytical X’Pert3 (±0.0025°, Malvern PANalytical Ltd., Worcestershire, UK). In preparation for XRD analysis, the wire sample segments were secured in resin. Once the resin had solidified, the resin-encased wire samples were ground down and polished to create a flat surface area suitable for XRD analysis. This process was carried out for wire samples 1 to 9 (see Table 2), as well as for the as-received untreated wires W1, W2, and W3.

Nanoindentation tests were performed on a Bruker HYSITRON TI Premier (Bruker, Karlsruhe, Germany) using a Berkovich Indenter to measure the variation in hardness (H) and elastic modulus (E) in the wires. In these load-controlled tests, a maximum load of 5000 µN was applied at a rate of 1000 µN/s and held for 2 s before unloading. A total of 400 indents were made on each sample within a 300 µm by 300 µm square grid, with 15 µm spacing between individual indents. The hardness (H) and elastic modulus (E) were calculated from the load-displacement data for all 400 points on each sample using the Oliver–Pharr method [28]. The hardness is found from

$$H=\frac{P_{max}}{A_{p}}$$

where $P_{max}$ is the maximum indentation load and $A_{p}$ is the projected area of contact, which are both taken directly from the indentation test data [29]. And elastic modulus is found from

$$E=\frac{1-{\left(v_{s}\right)}^{2}}{\frac{1}{E_{r}}-\frac{1-{\left(v_{i}\right)}^{2}}{E_{i}}}$$

where $E_{r}$ is the reduced modulus taken directly from the indentation test data, $E_{i}$ is the elastic modulus of the Berkovich indenter, and $v_{s}$ and $v_{i}$ are the Poisson’s ratios of NiTi and the inventor, respectively [29]. 

The elastic modulus E_i_ and Poisson’s ratio *v_i_* of the Berkovich indenter are taken as 1141 GPa and 0.07 in this study, and the Poisson’s ratio *v_s_* used is 0.33 [30,31].

## 3. Results and Discussion

### 3.1. Effect of Low-Temperature Aging on Actuator Stroke 

**Figure 3:**
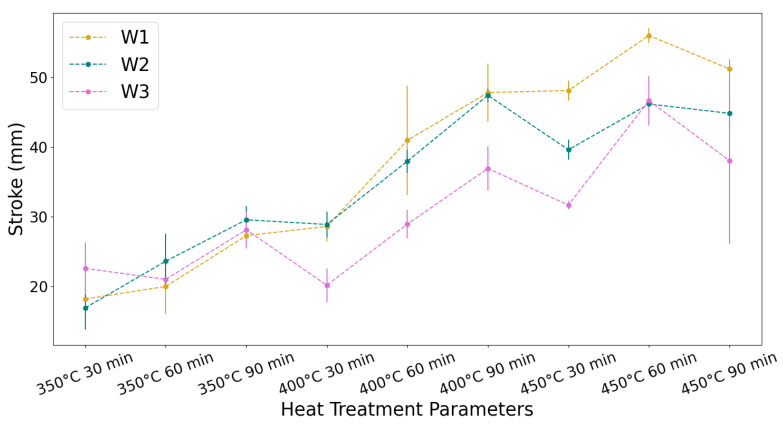
Graph of the average maximum stroke recorded from the heat-treated samples, n = 3.

The graph shown in Figure 3 presents the recorded displacements for each wire. The stroke achieved across all samples varies from the smallest stroke of 17 mm (±3.0 mm) for S10 (W2, 350 °C, 30 min) to the largest stroke of 56 mm (±1.0 mm) for S8 (W1, 450 °C, 60 min). There is a noticeable increase in actuator stroke with an increasing heat treatment temperature and time until 450 °C for 60 min; however, when the aging time is increased to 90 min at 450 °C, the actuator stroke decreases. 

Dislocations introduced during the wire-drawing process raise the stress required for plastic deformation, meaning that wires with a higher density of dislocations can store more energy during loading, which can be released on the reverse transformation. On the other hand, coherent Ni_4_Ti_3_ precipitates act in a similar way to the dislocations from cold work, acting as obstacles to dislocation motion, meaning the recovery stress of all samples increases with aging temperature and time. However, dislocations and precipitates also impede shape memory recovery and can prevent the material from undergoing the detwinning process or shape recovery during heating, which is necessary for the SME [24,25,26,27,28,29,30,31,32]. It has also been reported that dislocations impede both B19’ and R-phase transformation up to three times more than precipitates [33]; therefore, despite the increase in precipitation with increasing aging temperatures and times, the stroke increases with aging temperature and time due to increased defect annihilation.

The overall general increase in actuator stroke with an increased aging time and temperature is also attributed to the increased effectiveness of the shape-setting process. As the aging time and temperature increase, the shape setting improves, and the geometry of the wire becomes closer to the dimensions of the mandrel, with less springback on removal. It was also clear on visual inspection that the lower aging temperature of 350 °C is insufficient for shape setting. This agrees with similar findings in the literature, where Liu et al. [22] found that temperatures between 450 °C and 550 °C were best for shape setting of Ni_50.7_Ti_49.3_ wire stents, and Zhan et al. [32] found that the best aging for shape setting thin Ni_50.53_Ti_49.47_ sheets was at 30–60 min. 

The range of minimum and maximum stroke values observed across the tested samples varied among W1, W2, and W3. For W1, the stroke has a range of 38 mm, varying from 18.0 ± 1.5 mm (S1: 350 °C, 30 min) to 56.0 ± 1.0 mm (S8: 450 °C, 60 min). For W2, the stroke achieved has a range of 30 mm, varying from 17.0 ± 3.0 mm (S10: 350 °C, 30 min) to 47.0 ± 1.0 mm (S15: 450 °C, 60 min). In the case of W3, the stroke has a range of 27 mm, varying from 20.0 ± 2.5 mm (S22: 400 °C, 30 min) to 47.0 ± 3.5 mm (S26: 450 °C, 60 min). The variation between wire responses is due to the differences in their thermomechanical and heat treatment processing histories. Casati et al. [12] found that the drawing history carried out to achieve a given degree of cold work is crucial to the final properties of the wire. They found that the drawing history permanently alters the functional properties of the wire even after heat treatment and thermomechanical cycling. As the wires were all subject to the same degree of cold work but straight annealed at 480 °C, 510 °C, and 550 °C for W1, W2, and W3, respectively, the annihilation of defects would have been highest in W1 and lowest in W3.

At 350 °C, the aging conditions are not sufficient for shape setting; therefore, the stroke is primarily dictated by the degree of plastic deformation rather than shape memory recovery. For samples aged at 350 °C for 30 min, W3 has the highest stroke as it has the highest density of dislocations and thus the lowest plastic deformation. As the aging time increases to 60 and 90 min, precipitation of Ni_4_Ti_3_ occurs, which decreases both the plastic deformation and the shape memory recovery, resulting in W2 having the optimum balance between these effects. As the aging temperature increases to 400 °C and 450 °C, the aging conditions become sufficient for shape setting, and thus the stroke is primarily dictated by shape memory recovery. As W1 has the lowest density of dislocations, which impede shape memory recovery, W1 achieves the highest stroke at the aging temperatures of 400 °C and 450 °C. The decrease in stroke displacement for all wires aged for 90 min indicates that the density of precipitates at 90 min could be inhibiting the SME. The optimum aging parameter for actuator stroke displacement for all wires is 450 °C for 60 min. 

### 3.2. Effect of Low-Temperature Aging (350 °C to 450 °C) on Actuation Time

**Figure 4:**
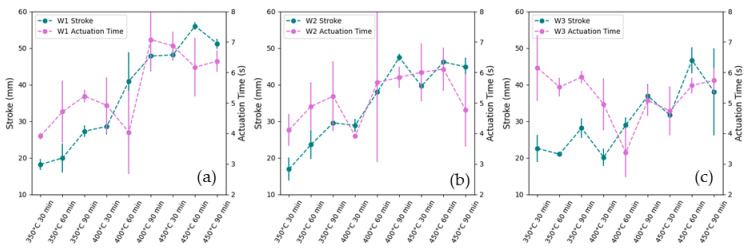
Graph of the actuation time and stroke for (**a**) W1, (**b**) W2, and (**c**) W3 achieved by heat-treated samples, n = 3.

From the results, the actuation time was found to vary between 3.4 and 7.1 s. At the low aging condition of 350 °C for 30 min, the actuation time increased from W1 to W3 (4.0 ± 0.1 s, 4.0 ± 0.5 s, and 6.0 ± 1.1 s for W1, W2, and W3, respectively), which may be attributed to the increase in A_f_ temperatures (42.72 °C, 46.15 °C, and 48.96 °C for W1, W2, and W3, respectively) requiring more time to complete actuation. However, as shown in Figure 4, there is no consistent trend in actuation time across all samples. This variability may be due to two competing factors: the increase in stroke and the decrease in A_f_ with higher aging temperatures. A larger stroke extends the actuator’s travel distance, increasing the actuation time, while a lower A_f_ reduces the time needed to reach the actuation temperature. Thus, the actuation time is determined by the interaction between A_f_ temperature and stroke.

**Figure 5:**
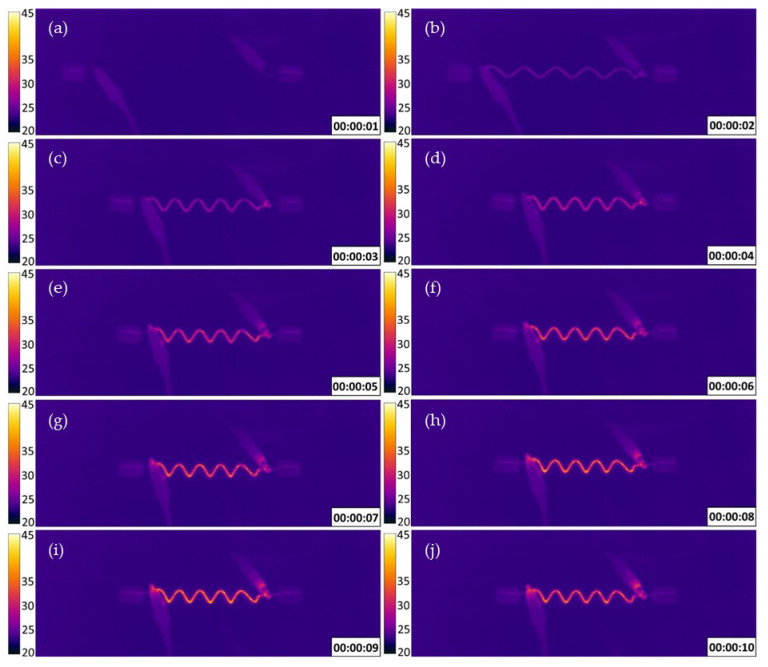
Thermal images (°C) and actuation timestamps (s) of sample 26 showing the effect over time of the current applied across the wire on the shape memory effect and temperature of the wire itself. After the current is applied at 0 s, (**a**,**b**) show the initial ‘dead time’ where the temperature of the wire is insufficient for shape recovery. At (**c**) the wire beings to heat and the length of the wire is reduced. In (**d**,**e**) the temperature of the wire continues to increase and the length decreases between 4 and 5 s. At 6 s (**f**) shape recovery is completed and the wire remains at the same length for the remaining time (**g**–**j**).

The thermal IR image data of S26 (450 °C, 60 min) during testing is shown in Figure 5. Figure 5a,b show the 2 s delay that occurred between the application of current and the wire’s corresponding heating and stroke response. This "dead time" is the duration required for the wire’s temperature to reach the phase-change temperature necessary to reverse martensite transformation [34]. Beyond this dead time, at 4 s, the wire’s temperature has become uniform along its length, and the majority of the shape recovery has occurred. At 6 s, shape recovery is complete, as shown in Figure 5d, which agrees with the results for sample 26 shown in Figure 4.

As rapid actuation is an important consideration in the functionality of actuators, further work is required on the improvement of actuation time, such as improved conductivity via alloying elements such as Cu and rapid heating pulse methods to reduce the time taken for the element to become uniformly heated and reduce loss of energy to the surroundings [35,36], and reduced thermal hysteresis (discussed in Section 3.3) to decrease the deadtime. 

### 3.3. Phase Transformation Temperatures and DSC Results 

#### 3.3.1. DSC Peak Analysis

The DSC curves of all samples tested in this study exhibit a symmetric multi-step transformation (MST). Upon heating, two peaks appear: the first is the B19’ to R-phase transformation and the second is the R-phase to B2 transformation. On cooling, two peaks are also observed, B2 to R-phase and R-phase to B19’. The second peak is broad and difficult to distinguish, preventing the identification of the B19’ finish (M_f_) or B’19 peak (M_p_), and in some cases, even the B19’ start (M_s_). It is common for the B19’ transformation peak to become broad and seem not to appear at all in NiTi with high levels of retained cold work [37]. In fact, the R-phase is associated with a large decrease in M_s_ due to internal stresses and dislocations increasing the barrier for martensite phase transformations, thereby facilitating the formation of the intermediate R-phase [38]. Additionally, symmetric R-phase MSTs can be uniquely characterised by a large disparity in peak spacing between heating and cooling [37].

R-phase transformations occur most frequently in Ni-rich NiTi and are often reported in the literature to be caused by defects induced by cold work and the presence of Ni_4_Ti_3_ precipitates, which increase the energy barrier to martensite formation, thus promoting the R-phase due to its lower activation energy [23,38,39,40]. The degree of cold work and annealing, however, can significantly influence the transformation [37].

**Figure 6:**
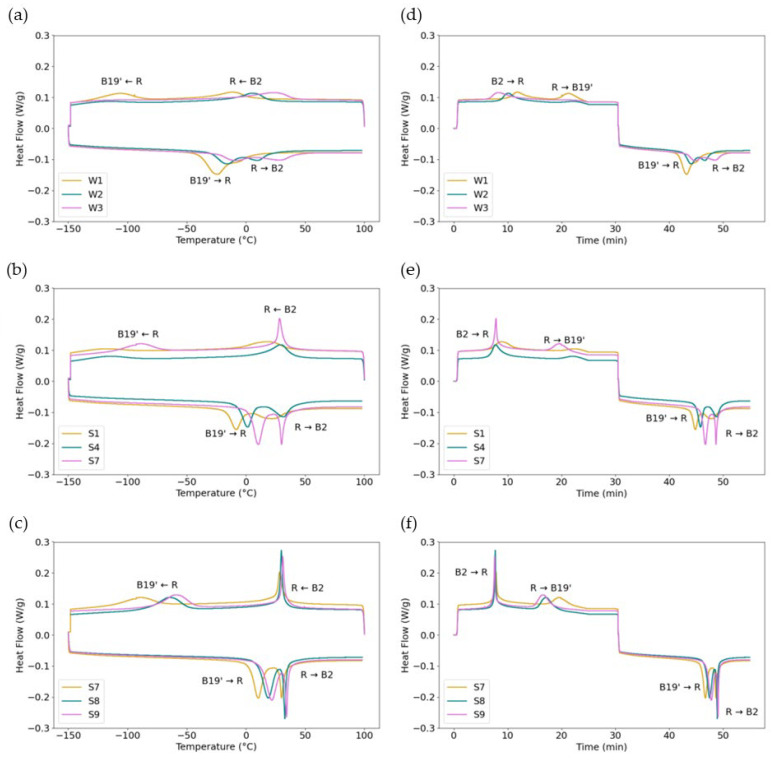
DSC curves of the as-received and untreated wires W1, W2, and W3 with (**a**) heat flow vs. temperature and (**d**) heat flow vs. time; S1, S4, and S7 with the same starting wire (W1) and heat treatment time (30 min) and different heat treatment temperatures of 350 °C, 400 °C, and 450 °C, respectively, with (**b**) heat flow vs. temperature and (**e**) heat flow vs. time; S7, S8, and S9 with the same starting wire (W1) and heat treatment temperature (450 °C) and different heat treatment times of 30, 60, and 90 min, respectively, with (**c**) heat flow vs. temperature and (**f**) heat flow vs. time.

Figure 6 shows three plots comparing selected DSC curves to determine the effect of different parameters on the phase transformation sequence. Figure 6a compares the as-received wires. The DSC curves of the as-received wires show broad transformation peaks on both cooling and heating. On heating, W1 shows two overlapping peaks that both broaden and separate from W1 to W2 to W3. The increased separation of the two peaks from W1 to W2 and W3 suggests an increase in lattice defects, suppressing phase transformation. On cooling, the two separate transformation peaks are present in W1, which broaden and separate from W1 to W3, such that the second peak seen in W1 is barely visible for W2 and disappears by W3. In fact, for W3, the second peak is not observed in the as-received sample or in samples heated at 350 °C for any length of time, or at 400 °C for 30 min. 

Figure 6b compares the effect of the heat treatment temperature using S1, S4, and S7, each from W1, and treated for 30 min at 350 °C, 400 °C, and 450 °C, respectively. There is a clear sharpening of peaks with increasing temperature across the samples for both heating and cooling curves. Additionally, the B19’→R and R→B2 peaks become closer with increasing temperature. Figure 6c compares the effect of the heat treatment time using S7, S8, and S9, all from W1, and treated at 450 °C for 30, 60, and 90 min, respectively. There is a substantial increase in peak sharpness between S7 aged at 30 min and sample 8 aged at 60 min, and a minor decrease from S8 to S9 aged at 90 min. However, the difference in sharpness between samples treated at different times is considerably less significant than the difference observed between samples treated at varying temperatures. The separation between B19’→R and R→B2 peaks for all aging times at 450 °C is considerably reduced from the as-received sample, indicating a reduction in dislocations from cold work. Still, there are two clear peaks visible, likely due to insufficient defect annihilation and the precipitation of secondary phases [41]. The broadening of DSC phase transformation peaks indicates the condition of stresses in the crystal. A broader peak indicates the presence of internal strains impeding the phase transformation caused by dislocations and precipitates, and the presence of stress-induced martensite [42,43,44]. Therefore, based on the DSC curves, the amount of residual stress-induced martensite and defects increases from W1 to W3. This indicates that an increasing aging temperature and time reduce lattice defects and stress-induced martensite, and the aging temperature has a more significant effect than the aging time.

#### 3.3.2. Transformation Temperatures

**Figure 7:**
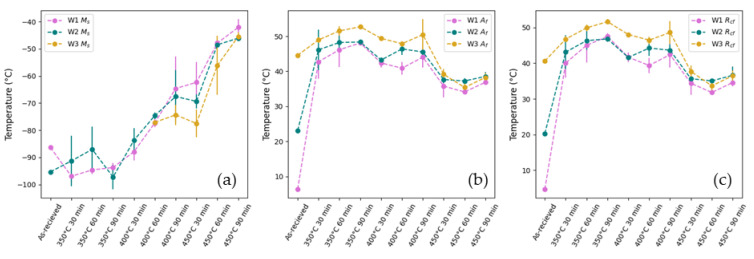
Graph of the (**a**) B19’ start temperature, (**b**) B2 finish temperature, and (**c**) R-phase finish temperature, n = 3.

The M_s_ temperature, A_f_ temperature, and cooling R-phase finish temperature (R_cf_) obtained from the DSC experiments are shown in Figure 7 below. The results show that A_f_ and R_cf_ increase and the M_s_ temperature decreases from the as-received W1 to W3. This variation in TTs between the as-received wires is due to the differences in their thermomechanical history. Cold work, such as wire drawing, introduces dislocations and residual stresses that inhibit the phase transformations, resulting in higher A_f_ temperatures, lower M_s_ temperatures, and stabilization of the R-phase. Annealing the wires reduces the number of dislocations and relieves the stresses, allowing phase transformation to occur more readily, thus decreasing A_f_ and R_cf_ temperature and increasing M_s_ temperature [11,41,43,45,46].

The M_s_ temperature shows a large increase with both aging temperature and time. To fabricate room-temperature SMA actuators using R-phase transformations, the M_s_ temperature must be kept below room temperature. Despite the large increase in the B19’ transformation temperatures compared to B2 and the R-phase, the aging conditions in this study maintain a low M_s_ temperature.

A significant reduction in the variation between W1, W2, and W3 of A_f_ and R_cf_ from the as-received wires to the heat-treated wires can be seen in Figure 7b,c, suggesting that the differences in microstructure amongst samples resulting from the thermomechanical processing were reduced through the further post-processing heat treatments.

The results also show a significant increase in A_f_ from the as-received wires to the samples aged at 350 °C for 30 min. Increases in the TTs of NiTi are commonly attributed to the precipitation of Ni-rich precipitates as they deplete Ni from the matrix, thus increasing A_f_. This initial increase in A_f_ after 30 min of aging may therefore be due to the rapid precipitation of Ni_4_Ti_3_ at shorter times. A similar trend was seen by both Liu et al. [22] and Drexel et al. [47], who report a rapid increase in A_f_ temperature owing to the high rate of Ni_4_Ti_3_ precipitation for samples annealed between 300 °C and 500 °C for 2 min to 30 min and increases only slightly at longer aging times. Additionally, as reported by Favier et al. [20], constraining the sample for shape setting results in faster aging kinetics due to the interaction between the internal stresses from Ni_4_Ti_3_ precipitates and the applied external stress during constrained aging, meaning that the saturation of precipitation could occur within the shorter aging times (<30 min) in this study.

There is a general decrease in the A_f_ temperature with an increasing aging temperature. While this may be assigned to a decrease in Ni_4_Ti_3_ precipitates, it is well reported in the literature that the optimum temperature for Ni_4_Ti_3_ precipitation occurs between 400 °C and 450 °C for Ni-rich NiTi. Therefore, it is likely that as the aging temperature increases, the dislocations and residual stress from cold work are more effectively removed while the continued precipitation of Ni_4_Ti_3_ prevents the A_f_ from returning to the pre-aged A_f_ for W1 and W2 [47].

For longer aging times, there is a slight increase in A_f_ at 350 °C, but no clear trend with aging time for higher aging temperatures. Kaya et al. [48] postulate that the increase in A_f_ with aging time is due to the influence of Ni_4_Ti_3_ precipitates on the martensite morphology. At lower aging times, Ni_4_Ti_3_ precipitates are expected to be small and closely spaced. This creates local stress fields in the alloy matrix, facilitating the nucleation of large martensite plates at lower temperatures. As the aging time increases, the volume fraction of precipitates remains unchanged, but the size of the precipitates increases. As the precipitates grow, the distance between them increases, reducing the effect of local stress fields on martensite nucleation, and allowing the change in Ni content of the matrix to become the driving force for changes in A_f_. The growth of Ni_4_Ti_3_ precipitates with time increases the Ni depletion, thereby increasing A_f_ [48]. At higher aging temperatures, there are more factors influencing the change in A_f_ with aging time due to more effective recovery of retained martensite and the rearrangement and annihilation of dislocations coupled with an increased volume of Ni_4_Ti_3_, which may account for the lack of correlation between A_f_ temp. and aging time at 400 °C and 450 °C.

#### 3.3.3. Thermal Hysteresis

Another characteristic of the R phase is a small thermal hysteresis of the B2-R phase transformation compared to that of the B2-B19’ transformation, which decreases with an increasing aging temperature [37,40]. This characteristic can be beneficial for applications in micro-actuators with a high working frequency [49]. Thermal hysteresis ‘ η ’ is defined as the temperature differential between the forward and reverse phase change transformations and can be calculated as the difference in temperature between the martensite peak or the R-phase peak on cooling and the austenite peak on heating [50]. Thermal hysteresis is attributed to the elastic misfit between phases and is generally lower in Ni-rich NiTi. However, the presence of dislocations, defects, residual stress, or chemical heterogeneity in the material, all of which are inherent to cold working, can greatly alter thermal hysteresis by either inhibiting or promoting the phase transformations [50].

**Figure 8:**
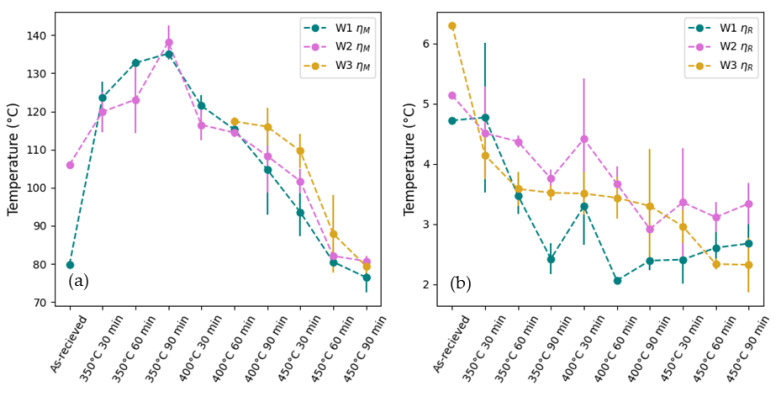
Graph of (**a**) B2-B19’ thermal hysteresis and (**b**) B2-R thermal hysteresis of the as-received and heat-treated samples calculated from DSC, n = 3.

Figure 8 compares the thermal hysteresis of the B19’ to B2 transformation ( $\eta_{M}$ ), defined as the difference between the B2 peak temperature (A_p_) and M_s_ (note that as mentioned, the broad undefined nature of the B19’ peaks in this study meant that M_p_ could not be identified and instead $\eta_{M}$ was calculated as the difference in temperature between M_s_ and A_p_) and the thermal hysteresis of the R-phase to B2 transformation ( $\eta_{R}$ ), defined as the difference between A_p_ and the R-phase peak on cooling (R_cp_). 

The results of this study, presented in Figure 8a, revealed a significant level of $\eta_{M}$ in the wires, ranging from approximately 76.44 ± 3.87 °C to 138.19 ± 4.42 °C. This level of thermal hysteresis is not uncommon in wire-drawn alloys. For example, in a study by Choi et al. [45], inducing a prestrain of 7% during wire drawing of NiTiNb alloys increased the A_f_ temperature from 22.02 °C to 139.18 °C and increased the thermal hysteresis from 31.59 °C to 122.5 °C. The results show that $\eta_{M}$ for the as-received wires was lowest for W1 and highest for W3, although the exact value of $\eta_{M}$ could not be calculated as the M_s_ temperature could not be identified due to the broad nature and low temperature of the martensite transformation. Among the aged samples, the results show a decrease in $\eta_{M}$ with an increasing aging temperature. The residual stresses and dislocations introduced during wire drawing can pin or stabilise the martensite, increasing the energy barrier for phase transformations and thus increasing thermal hysteresis. As the annealing temperature increases, the residual stress and dislocations decrease, facilitating phase transformations and reducing hysteresis [41,51]. The results also show that hysteresis increases with time for samples heated at 350 °C and decreases with time for samples heated at 400 °C and 450 °C. As discussed in Section 3.1, precipitates have a similar effect to dislocations in terms of inhibiting phase transformation. These results indicate that 350 °C is insufficient for reducing dislocations and residual stress but sufficient for the precipitation of Ni_4_Ti_3_. At 350 °C, increasing the aging time results in an increase in Ni_4_Ti_3_ precipitates, causing an increase in hysteresis. As the aging temperature is increased, it becomes sufficient for the removal of residual stress and dislocations, which becomes the deciding factor for hysteresis. Thus, hysteresis decreases with aging time for aging temperatures of 400 °C and 450 °C, despite the continued precipitation of Ni_4_Ti_3_.

The results for $\eta_{R}\;$ presented in Figure 8b show a significantly smaller hysteresis compared to $\eta_{M}$ , ranging from 2.06 ± 0.01 °C to 4.77 ± 1.24 °C. As with $\eta_{M}$ , W1 has the smallest $\eta_{R}$ of the as-received wires (4.79 °C), followed by W2 (5.14 °C) and then W3 (6.30 °C). Among the aged samples, there is a general decrease in $\eta_{R}$ with aging temperature. Variations in $\eta_{R}$ are much smaller than those of $\eta_{M}$ due to the relatively smaller variation in A_p_ and R_cp_ temperatures compared to the M_s_ temperatures.

### 3.4. Phase and Microstructural Analysis 

**Figure 9:**
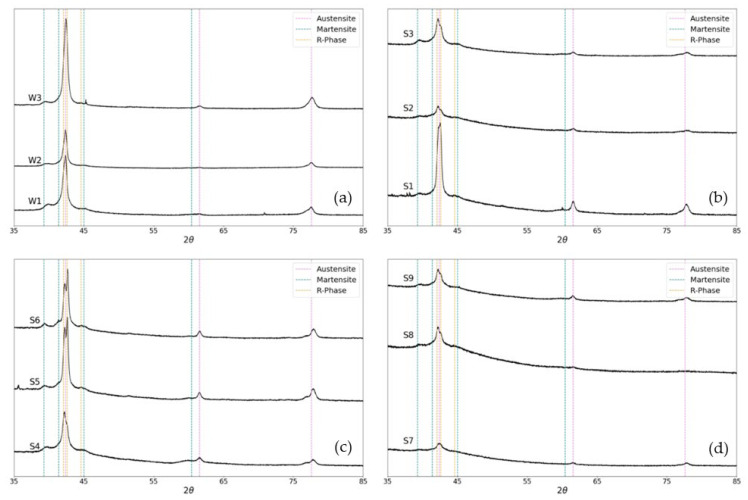
XRD patterns showing the presence of the austenite, martensite, and R-phase in (**a**) W1, W2, and W3 before heat treatment with sample 6 (W1, 400 °C, 90 min) as a control, (**b**) samples 1–3 (W1, 350 °C, for 30, 60, and 90 min, respectively) with sample 6 as a control, (**c**) sample 4–6 (W1, 400 °C, for 30, 60, and 90 min, respectively), and (**d**) samples 7–9 (W1, 450 °C, for 30, 60, and 90 min, respectively) with sample 6 as a control.

NiTi wires with the same chemical composition but drastically different microstructures have been reported in the literature as a consequence of the different degrees of cold work and annealing [52]. Figure 9a–d show the XRD patterns of as-received W1, W2, and W3 and the heat-treated wires S1–S9. Due to the wire’s small diameter of 0.46 mm, achieving a sufficiently large surface area of material to obtain clear peaks was not possible for all samples. The primary phases identified in these samples were austenite, martensite, and R-phase. In the as-received wires, there is a single peak at approximately 2ϴ = 42°, which splits into two peaks in the aged samples. The second peak at approximately 2ϴ = 39° represents the R-phase. This therefore indicated that aging stabilised the R-phase. As the density of defects decreases from the as-received samples to the aged samples, it can be concluded that the stabilisation of the R-phase is caused by the precipitation of Ni_4_Ti_3_.

### 3.5. Mechanical Properties

Table 3 provides a summary of the average values obtained from the nanoindentation tests, which represent 400 points of measurements on each sample. The hardness (H) and elastic modulus (E) were calculated from the nanoindentation results using the Oliver–Pharr method [28].

The elastic modulus is a measure of the stiffness of a material and its ability to resist elastic deformation or deflection under an applied force and is dependent on factors including the density and size of precipitates, grain size, defects, and volume fraction of phases present [52,53]. It is often reported in the literature that the B19’ phase has a lower elastic modulus than the B2 phase and that the elastic modulus typically ranges from 18.45 GPa to 45 GPa for B19’ and 41 GPa to 75 GPa for B2 [53,54,55]. However, a wide range of values for both phases has been reported in the literature. For example, Obeidi [56] reported an elastic modulus of between 62 GPa and 79.5 GPa for B19’, and Huang et al. [57] reported an elastic modulus of 73.7 ± 4.2 for B19’ and 80.1 GPa for B2. As well as the main B2 and B19’ phases, Wagner and Windl [58] reported that the elastic modulus of the pure B2 phase increased from 71 GPa to 108 GPa due to the precipitation of Ni_4_Ti_3_, and Yang et al. [52] reported an increase from 118 GPa in the B2 phase to 163 GPa with the addition of Ni_4_Ti_3_ precipitates. Fine, densely distributed Ni_4_Ti_3_ precipitates inhibit dislocation motion, which increases the elastic modulus. An increase in density of precipitates increases this effect further; however, as the size of the precipitates increases, they become incoherent and the elastic modulus decreases [32,59]. In addition to the volume fraction of phases present and the precipitation of Ni_4_Ti_3_, it has been reported that aging causes an increase in grain size, and that for grain sizes of less than 60 nm, the elastic modulus decreases with increasing grain size due to the larger grains decreasing the stress required to form stress-induced martensite [39,60].

W1 and W2 show an initial increase in E for the aging condition of 350 °C for 90 min, followed by a decrease for the aging condition of 450 °C for 60 min. For W3, E decreases for the aging condition of 350 °C for 90 min and increases for the aging condition of 450 °C for 60 min. For W1 and W2, the increase in E from the as-received samples to those aged at 350 °C for 90 min may be due to the precipitation of Ni_4_Ti_3_, which increases E. Conversely, the decrease in E for samples aged at 450 °C for 60 min may be attributed to a decrease in the density of defects. The decrease in E from the as-received samples to those aged at 350 °C for 90 min in W3 may be due to the increase in volume fraction of the lower elastic modulus B19’ phase. As the results of this study show that this sample was found to have the highest TTs and the most martensitic microstructure, the increase in E for the aging condition of 450 °C for 60 min may be due to the increase in the volume fraction of the higher-elastic-modulus B2 phase, as TTs decrease and the microstructure becomes more austenitic.

The hardness of the NiTi wire is a consequence of the microstructure owing to its thermomechanical processing and post-process aging. Frick et al. found that hardness in cold-draw Ni_50.9_Ti_48.1_ wires is primarily dependent on dislocation density, and that aging between 350 °C and 550 °C reduces the hardness due to the annihilation of dislocations [61]. Morawiec at al. found that hardness increased with cold rolling due to an increase in dislocation density. They also found that hardness decreases linearly with an increasing aging temperature above 400 °C. They attributed the decrease in hardness between 400 °C and 500 °C to the reduction in dislocation density; however, they found that although the hardness continues to decrease at temperatures above 500 °C, there is no further decrease in dislocation density. The decrease at temperatures above 500 °C is therefore said to be due to some additional factors [43].

The precipitation of Ni_4_Ti_3_ causes an increase in hardness and decrease in ductility due to precipitation hardening. Hardness has a complex dependence on precipitate formation and is significantly affected by the size, volume, and interparticle spacing of the precipitates [59,60]. According to Tahaei et al., the hardness and resistance to martensite transformation are determined by the size of Ni_4_Ti_3_ precipitates, with the researchers finding that hardness is highest when precipitates are around 10 nm, and hardness decreases as the precipitate size increases [60]. This is because small precipitates are coherent with the matrix, making them more effective at inhibiting dislocation motion. As the aging temperature and time increase, the precipitates grow and coarsen, becoming incoherent with the matrix, and thus decreasing hardness [61]. For example, Kaya et al. found that hardness was relatively unchanged by increasing the aging temperature from 400 °C to 500 °C, but decreased with an increasing aging time from 1 h to 24 h, with the researchers attributing this to an increase in precipitation size and interparticle distance [48]. Increasing aging temperatures can also reduce hardness if the temperature exceeds the conditions for Ni_4_Ti_3_ precipitation. For example, Drexel et al. reported that aging Ni_50.8_Ti_48.2_ wires between 350 °C and 450 °C resulted in the precipitation of Ni_4_Ti_3_, but aging at higher temperatures caused the precipitates to dissolve [47].

The results show a general decrease in the average nano-hardness of the as-received wires with aging. The presence of precipitates or a high density of dislocations essentially affects the hardness of NiTi in the same way. In either case, they inhibit plastic flow, which increases hardness. The difference is that dislocations are reduced during aging while precipitates are created. Therefore, the effect of aging on the hardness of NiTi is a function of two opposing processes – the annihilation of dislocations and growth of Ni_4_Ti_3_ precipitates [61]. Based on the literature and the nanoindentation results of this study, the dislocations and retained martensite introduced during the wire-drawing process contributed to the relatively high hardness values of the as-received wires (4.17 GPa, 4.34 GPa, and 4.24 GPa for W1, W2, and W3, respectively). The overall decrease in hardness with an increasing aging temperature suggests that hardness is determined by the dislocation density, which is in agreement with the findings of Frick et al. mentioned above [61]. As the aging temperature increases, the dislocation density decreases, leading to a decrease in hardness.

**Figure 10:**
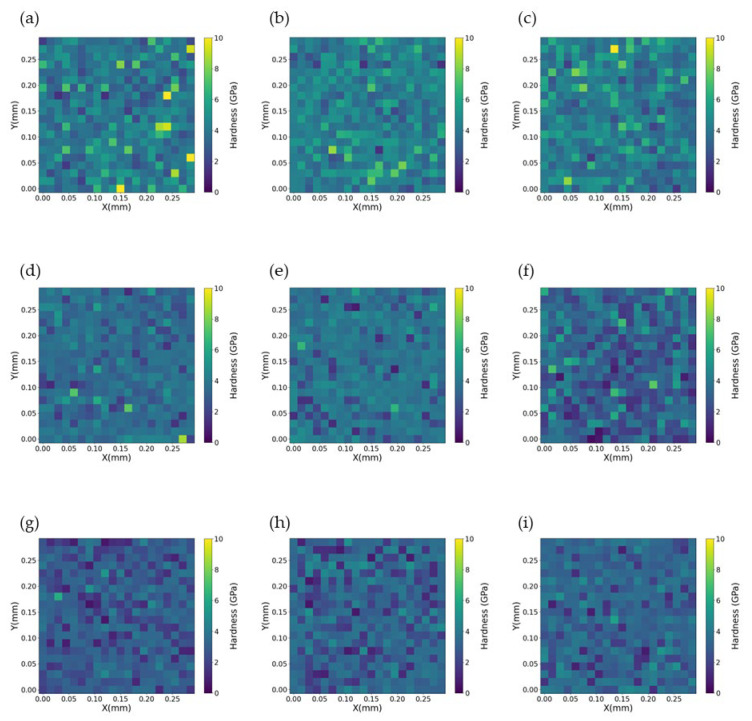
Hardness maps illustrating the measured nano-hardness values at each of the 400 indentation points on each sample: (**a**) W1 (as-received), (**b**) W2 (as-received), (**c**) W3 (as-received), (**d**) S3 (W1, 350 °C, 90 min), (**e**) S12 (W2, 350 °C, 90 min), (**f**) S21 (W3, 350 °C, 90 min), (**g**) S8 (W1, 450 °C, 60 min), (**h**) S17 (W2, 450 °C, 60 min), and (**i**) S26 (W3, 450 °C, 60 min).

The distribution of the hardness results for all 400 data points on each sample is visually illustrated in Figure 10. The hardness values for the as-received W1, W2, and W3 were distributed from 1 GPa to 10 GPa, with the main peaks at 3.7 GPa, 4.6 GPa, and 4.0 GPa, respectively. The distribution of hardness values decreased from the as-received wires to the aged wires, and among the aged samples, the hardness distribution decreased with an increased aging temperature. The hardness for samples aged at 350 °C for 90 min was distributed from 0.75 GPa to 8.75 GPa. Main peaks were identified at 3.7 GPa and 4.2 GPa for S3 (W1) and S12 (W2), respectively. The distribution is wider for S21 (W3) and appears to have two main peaks, around 2.4 GPa and 3.8 GPa. The hardness for samples aged at 450 °C for 60 min was distributed from 0.5 GPa to 6.25 GPa, with main peaks at 3.3 GPa, 3.6 GPa, and 3.3 GPa for S8 (W1), S72 (W2), and S26 (W3), respectively. Hardness distribution values within the same sample indicate a heterogenous distribution of precipitates and stress-induced B19’ phase [39]. The high variability in the nano-hardness in the as-received wires therefore indicates a high degree of stress and phase variation owing to the wire-drawing process. The decrease in distribution with an increased aging temperature indicates a reduction in stress-induced martensite and lattice defects from the wire-drawing process and a more homogeneous distribution of precipitates. The two main peaks for S21 could represent two distinct phases in the material, potentially due to a higher degree of retained martensite or defects.

## 4. Conclusions

This study investigated the impact of thermomechanical processing on the actuation performance of Ni_50.9_Ti_49.1_ electromechanical helical spring actuators. The primary focus was on the effects of the wire-drawing history and heat treatment parameters, including straight annealing and shape-setting conditions, on the stroke, actuation time, and transformation temperatures of the Ni-rich nitinol springs.

The findings highlight that the final actuator properties are influenced by a combination of dislocation annihilation, Ni_4_Ti_3_ precipitation, and the thermomechanical processing history. It was observed that an increasing aging temperature and duration improved the actuator stroke up to an optimal condition, beyond which excessive precipitation inhibited shape memory recovery. The optimal processing parameters for maximizing actuator stroke while maintaining low hysteresis were identified as straight annealing at 480 °C, followed by shape setting at 450 °C for 60 min. These conditions achieved the best balance between defect annihilation and precipitation hardening, yielding a high-performing actuator with an A_f_ temperature of 34.11 ± 0.34 °C, a stroke of 56.0 ± 1.0 mm, a thermal hysteresis of 2.68 ± 0.26 °C, and an actuation time of 6 ± 1 s.

Unlike conventional NiTi actuators that rely on martensite transformation, this study investigates Ni_50.9_Ti_49.1_ helical spring actuators operated through an R-phase transformation. The stabilization of the R-phase due to controlled thermomechanical processing results in lower hysteresis, making it particularly advantageous for applications requiring precise actuation. Additionally, while Ti-rich NiTi is typically preferred for actuator applications due to its superior shape memory effect, this study shows that Ni-rich NiTi can be effectively tailored through thermomechanical treatments to achieve a comparable or even superior actuation performance, with the added benefits of reduced thermal hysteresis.

Microstructural and phase transformation analyses confirmed that the Ni_4_Ti_3_ precipitates played a crucial role in stabilizing the R-phase, which contributed to the observed actuation behaviour. Differential Scanning Calorimetry (DSC) results demonstrated a general reduction in transformation temperature hysteresis with an increasing heat treatment temperature. Furthermore, nanoindentation analysis revealed a decrease in hardness with an increasing aging temperature, indicating effective dislocation recovery and a transition toward a more homogeneous microstructure.

This study provides valuable insights into the processing–structure–property relationships in Ni-rich nitinol actuators, emphasizing the potential for tuning thermomechanical treatments to optimise actuator performance. Future work should explore alternative alloying strategies and advanced processing techniques, such as rapid heating pulse methods, to further enhance the actuation speed and efficiency.

## Figures and Tables

**Table 1:** DoE model of three parameters at three levels.

**Level**	**−1**	**0**	**+1**
**Starting Wire**	W1	W2	W3
**Treatment Temperature (** **°C** **)**	350	400	450
**Treatment Time (min)**	30	60	90

**Table 2:** Full table of the 27 process parameters used in this study.

Sample Name	Starting Wire	Heat Treatment Temperature (°C)	Heat Treatment Time (min)
S1	W1	350	30
S2	W1	350	60
S3	W1	350	90
S4	W1	400	30
S5	W1	400	60
S6	W1	400	90
S7	W1	450	30
S8	W1	450	60
S9	W1	450	90
S10	W2	350	30
S11	W2	350	60
S12	W2	350	90
S13	W2	400	30
S14	W2	400	60
S15	W2	400	90
S16	W2	450	30
S17	W2	450	60
S18	W2	450	90
S19	W3	350	30
S20	W3	350	60
S21	W3	350	90
S22	W3	400	30
S23	W3	400	60
S24	W3	400	90
S25	W3	450	30
S26	W3	450	60
S27	W3	450	90

**Table 3:** Average elastic modulus (E) and hardness (H) calculated from nanoindentation results, n = 400.

Sample	E (GPa)	H (GPa)
W1 (as-received)	55.8 ± 12.5	4.17 ± 1.36
S3 (W1, 350 °C, 90 min)	65.5 ± 10.5	3.52 ± 0.83
S8 (W1, 450 °C, 60 min)	60.7 ± 11.5	2.79 ± 0.84
W2 (as-received)	59.20 ± 8.81	4.34 ± 1.01
S12 (W2, 350 °C, 90 min)	64.80 ± 9.44	3.73 ± 0.84
S17 (W2, 450 °C, 60 min)	60.4 ± 12.6	3.06 ± 0.96
W3 (as-received)	59.9 ± 10.2	4.24 ± 1.13
S21 (W3, 350 °C, 90 min)	56.1 ± 14.3	3.16 ± 1.18
S26 (W3, 450 °C, 60 min)	60.5 ± 10.5	3.20 ± 0.82

## Data Availability

The original contributions presented in this study are included in the article. Further inquiries can be directed to the corresponding author.
