# Correction: Ryan Murphy et al. Investigating the Effect of Wire Drawing and Heat Treatment on the Response of Ni_50.9_Ti_49.1_ R-Phase Actuators. *Materials* 2025, *18*, 4931

**DOI:** 10.3390/ma19010040

**Published:** 2025-12-22

**Authors:** Josephine Ryan Murphy, Muhannad Ahmed Obeidi, Inam Ul Ahad, Dermot Brabazon

**Affiliations:** 1 School of Mechanical and Manufacturing Engineering, Dublin City University, 9 Dublin, Ireland; obeidimuhannad@gmail.com (M.A.O.); inamul.ahad@dcu.ie (I.U.A.); dermot.brabazon@dcu.ie (D.B.); 2 I-Form Advanced Manufacturing Centre Research, Dublin City University, 9 Dublin, Ireland; 3 Advanced Metallic Systems Centre for Doctoral Training, School of Mechanical & Manufacturing Engineering, Dublin City University, 9 Dublin, Ireland

In the original publication [1], there were errors regarding the affiliations 2 and 3. The corrected affiliations should be as follows:

2 I-Form Advanced Manufacturing Centre Research, Dublin City University, 9 Dublin, Ireland
3 Advanced Metallic Systems Centre for Doctoral Training, School of Mechanical & Manufacturing Engineering, Dublin City University, 9 Dublin, Ireland

Additionally, the authors Muhannad Ahmed Obeidi, Inam Ul Ahad and Dermot Brabazon should also be marked with affiliation 3.

The authors state that the scientific conclusions are unaffected. This correction was approved by the Academic Editor. The original publication has also been updated.
